# Adult Reactive Infectious Mucocutaneous Eruption (RIME) from Concurrent Adenovirus and *Mycoplasma pneumoniae* Infections

**DOI:** 10.1007/s11606-025-09827-6

**Published:** 2025-09-10

**Authors:** Jeffrey Li, Sasan D. Noveir, Natalie Bouri, Kyle Cheng, Inderpreet Saini

**Affiliations:** 1 David Geffen School of Medicine at University of California Los Angeles, Los Angeles, CA USA; 2 Department of Medicine, Stanford University School of Medicine, Stanford, CA USA; 3 Department of Medicine, David Geffen School of Medicine at University of California Los Angeles, Los Angeles, CA USA; 4 Division of Dermatology, Department of Medicine, David Geffen School of Medicine at University of California Los Angeles, Los Angeles, CA USA

## INTRODUCTION

*Mycoplasma pneumoniae* is a well-known cause of atypical pneumonia but can also lead to various dermatologic manifestations, including mucocutaneous erosive eruptions.^1,2^ In 2015, the term *Mycoplasma*-induced rash and mucositis (MIRM) was introduced to describe this clinical entity, with typical presentations including vesiculobullous lesions, hemorrhagic crusting of the lips, erosions on the tongue and buccal mucosa, and purulent bilateral conjunctivitis.^2^ More recently, the umbrella term “reactive infectious mucocutaneous eruption (RIME)” was introduced to more broadly describe post-infectious mucocutaneous pathology caused by any infection including *Mycoplasma pneumonia* (MIRM), *Chlamydia pneumoniae*, and viruses such as adenovirus and enteroviruses.^3,4^

The diagnostic challenge posed by these novel definitions and the lack of a standardized treatment for MIRM underscore the importance of identifying the infectious agent. While rare, cases can be further complicated by *Mycoplasma pneumonia* co-infections with other RIME-inducing agents.^5–7^ Although RIME is primarily described in pediatric patients, adult cases do occur. This report uniquely describes an adult initially diagnosed with adenovirus-associated RIME, later found to have concurrent MIRM after worsening symptoms despite initial corticosteroid treatment, and successfully managed with azithromycin, cyclosporine, and etanercept.

## CASE DESCRIPTION

**Figure 1:**
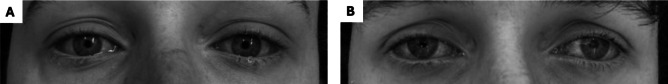
Conjunctivitis on first hospital admission (**A**) and on readmission 3 days later (**B**).

**Figure 2:**
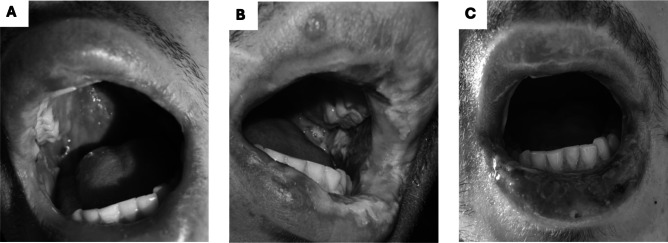
Shallow erythematous erosions on buccal mucosa and mucosal lips on first hospital admission (**A**). Worsening erosions with hemorrhagic yellow crusting on readmission 3 days later (**B**). Improved mucositis on the day of discharge (**C**).

A 20-year-old Caucasian male with no significant past medical history presented to the emergency department with conjunctivitis, photophobia, and painful oral ulcers for 3 days. He had been started on levofloxacin around the same time for treatment of pneumonia, having experienced 2 weeks of cough and dyspnea, with a chest X-ray demonstrating a lobar infiltrate. On admission, the patient’s vitals were normal, and labs were notable for a WBC count of 16.73 × 10^3^/μL with an elevated neutrophil count of 14.04 × 10^3^/μL. Physical exam revealed conjunctival injection (Fig. 1A) and erosions on the buccal mucosa and mucosal lips (Fig. 2A). Levofloxacin was discontinued due to concern for SJS. Respiratory panel was ordered and revealed positive adenovirus PCR. *Mycoplasma* serology was ordered but had not resulted. Given the lack of cutaneous skin blistering and mucositis-dominant findings, along with a positive adenovirus PCR result, a diagnosis of adenovirus-induced RIME was favored over SJS, and the patient was discharged with a methylprednisolone 4-mg dose pack.

**Figure 3:**
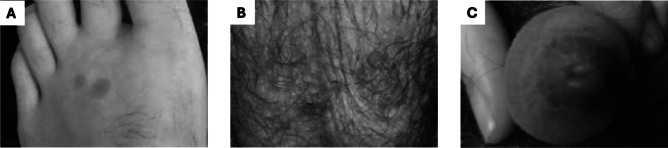
Erythematous targetoid papules on the dorsal foot (**A**), scrotum (**B**), and urethral meatus (**C**) after re-admission to the hospital 3 days later.

However, over the next 3 days, while his respiratory symptoms improved, the patient noticed increasing number of oral lesions, worsening bilateral vision with ocular pain, and development of genitourinary ulcers. At his outpatient ophthalmology follow-up, large conjunctival defects extending the entire length of the inferior bulbar conjunctiva were found, prompting a return to the emergency department. On readmission, he reported a weight loss of 10 pounds due to poor oral intake from severe ulcer-induced odynophagia. Clinical examination revealed bilateral conjunctivitis (Fig. 1B) as well as significant erosions on his buccal mucosa and mucosal lips with hemorrhagic yellow crusting (Fig. 2B). There was also new and progressing round erythematous macules on his bilateral feet (Fig. 3A), forearm, thigh, scrotum (Fig. 3B), and urethral meatus (Fig. 3C) affecting about 2% of his total body surface area. Labs showed a WBC of 12.04 × 10^3^/μL.

He was initially started on intravenous methylprednisolone 4 mg twice daily and underwent urgent bilateral amniotic membrane transplantation to address his conjunctival defects. The *Mycoplasma pneumoniae* serology from the prior hospitalization returned positive (IgG 0.37, IgM 0.80), consistent with MIRM. While his leukocytosis resolved the following day, his odynophagia worsened, and physical exam showed progressive oral mucositis. He was subsequently started on a 5-day course of azithromycin (500 mg day 1, followed by 250 mg for 4 days daily), cyclosporine 1.0 mg/kg twice daily, and received one dose of etanercept 50 mg. Adjunctive pain management, including miracle mouthwash (diphenhydramine-nystatin-magnesium hydroxide) for oral lesions and triamcinolone 0.1% ointment for genital lesions, was provided for symptom control. After significant improvement, including healing of oral erosions (Fig. 2C), resolution of skin papules, and restored ability to tolerate oral intake, he was discharged 4 days later on an oral cyclosporine taper (150 mg, 100 mg, 50 mg twice daily for 5 days each for a 15-day course) and triamcinolone 0.1% ointment.

At a dermatology follow-up 5 days post-discharge, his oral mucositis and genital erosions resolved. At an ophthalmology follow-up at 10 days and 1-month post-discharge, there was no evidence of symblepharon or other ocular complications. During a primary care evaluation 2 months post-discharge, the patient was asymptomatic, had regained weight, and at a follow-up approximately 1-year post-hospitalization, reported no long-term sequelae.

## DISCUSSION

While MIRM was historically grouped with erythema multiforme (EM), Stevens-Johnson syndrome (SJS), and toxic epidermal necrolysis (TEN), differences in its pathophysiology and management have led to its recognition as a distinct entity.^2^ MIRM is defined as erosions involving less than 10% total body surface area, affecting at least two mucosal sites, and with evidence of atypical pneumonia.^2^ Although its exact pathophysiology remains unclear, it is thought to involve a dysregulated immune response to the infectious trigger, with proposed mechanisms including immune complex–mediated vascular injury, cytotoxic T cell–mediated epithelial damage, and/or antibody-mediated processes.^8,9^

Now classified within the broader umbrella of respiratory infection–associated mucositis (RIME), MIRM is typically more common in children—with a review of 202 published cases revealing a mean age of 12 years—whereas SJS and TEN are more commonly diagnosed in adults.^2,10^ This predominance of pediatric cases has even prompted some experts to suggest incorporating young age into the diagnostic criteria for MIRM.^2,10^ Nonetheless, although uncommon, adult cases do occur, and this report underscores the importance of considering both RIME and SJS/TEN in instances of acute mucocutaneous erosive eruptions, regardless of patient age.

A recent or concurrent history of respiratory infection significantly raises suspicion for RIME. However, patients with atypical pneumonia, such as that caused by *Mycoplasma pneumoniae*, are often treated empirically with beta-lactams or fluoroquinolones, which can inadvertently trigger mucocutaneous lesions that resemble those of SJS. In these cases, mucosal-dominant findings with minimal skin involvement should prompt clinicians to consider RIME over SJS/TEN, which are characterized by more extensive skin involvement.

This case presented an additional diagnostic challenge due to the initial positive test for adenovirus, with the subsequent discovery of a concurrent *Mycoplasma pneumoniae* infection only after the patient's condition worsened despite initial steroid treatment. In the literature, while there is a growing body of pediatric cases describing single-infection-induced RIME, primarily MIRM, there are very few cases of RIME from co-infection. Limited reports include one case of MIRM with respiratory syncytial virus, four cases of recurrent RIME from MIRM with group A streptococcus, influenza A/B, and SARS-CoV-2 co-infections, and one recent case of MIRM complicated by herpes simplex virus dissemination.^5–7,11^ Although adenovirus infection alone has been shown to induce RIME in children, there are no reports of adenovirus co-infection with MIRM or any other infectious agent.^12^ In two recent reviews, which also evaluated adult cases, the reported adult RIME cases have been single-agent and primarily MIRM.^13,14^ Therefore, our case represents the first described instance of concurrent adenovirus and *Mycoplasma pneumoniae* infections contributing to RIME in an adult. This underscores the importance of expanding the diagnostic workup for mucocutaneous eruptions to include possible co-infections, which may impact prognosis and treatment.

Initial management of RIME often overlaps with that of suspected SJS/TEN due to the difficulty in distinguishing between these conditions in the acute care setting. For both conditions, supportive care—including hydration, pain control, and wound care—remains the cornerstone of treatment. Systemic steroids are used infrequently and typically only on a case-by-case basis.^2,15^ In contrast, growing evidence supports cyclosporine as an effective adjunctive therapy to control inflammation.^16^ Additionally, a small retrospective cohort study of six hospitalized pediatric patients with RIME suggested that a single dose of subcutaneous etanercept could halt progression, decrease pain, and reduce the length of hospital stay.^17^ A recent meta-analysis of patients with *Mycoplasma pneumoniae* revealed elevated serum TNF-α levels, suggesting that anti-TNF treatments like etanercept may be more responsive in MIRM compared to other causes of RIME.^18^ In cases of moderate-to-severe SJS-TEN, etanercept has also been shown to enhance re-epithelialization compared to corticosteroids, indicating its potential broad utility in severe mucocutaneous erosive eruptions.^19^

Although patients with MIRM are often empirically treated with antibiotics for atypical pneumonia, there is no clear consensus on whether antibiotic treatment shortens the course of the disease. Notably, in this case, the patient’s condition deteriorated during initial treatment of purported adenovirus-induced RIME with steroids, and only showed improvement after the addition of azithromycin and immunomodulators. This patient’s worsening condition without atypical antibiotic coverage suggests that reducing *Mycoplasma* organisms plays a role in managing the cutaneous symptoms of MIRM.^20^ This observation supports the diagnostic importance of identifying *Mycoplasma* and the potential therapeutic role of targeted antibiotic treatment.

Prompt consultation with dermatology and ophthalmology specialists is essential for early recognition and tailored management of the patient’s mucocutaneous symptoms. Interdisciplinary discussion with dermatology is critical for distinguishing between RIME and SJS/TEN, as well as guiding adjunctive treatment with agents like cyclosporine and etanercept. Ophthalmology involvement is important in managing ophthalmic complications, such as assessing significant conjunctival defects in this patient and minimizing the risk of long-term ocular complications through timely amniotic membrane transplantation. Given the various clinical challenges associated with RIME/MIRM, prompt specialty consultations should be considered standard in suspected cases, particularly those with progressively worsening mucosal involvement.

## CONCLUSION

This case of RIME due to concurrent *Mycoplasma pneumoniae* and adenovirus infections underscores the diagnostic and therapeutic complexities associated with differentiating the etiology of mucocutaneous eruptions. Clinicians should be attentive to mucositis-predominant findings in RIME as opposed to the cutaneous predominance seen in SJS/TEN, especially when initial treatment for atypical pneumonia in these patients might include SJS/TEN-associated antibiotics. Our case further emphasizes the importance of identifying the microbial cause of RIME, as patients with MIRM, even in the setting of co-infection, may benefit from targeted treatment with azithromycin and etanercept. However, further research is needed to better characterize the differences in management between MIRM and other causes of RIME. Prompt consultation with dermatology and ophthalmology facilitated early diagnosis and effective management in this case, which likely contributed to the patient’s positive outcome. Although RIME/MIRM predominantly affects pediatric patients, internists should remain vigilant in considering it within their differential diagnoses for mucocutaneous diseases and understanding its distinct diagnostic and management nuances.

## Data Availability

Data sharing is not applicable to this article as no datasets were generated or analyzed during the current study.
